# Setting the stage for health: Salutogenesis in midwifery professional knowledge in three European countries

**DOI:** 10.3402/qhw.v11.33155

**Published:** 2016-12-13

**Authors:** Claudia Meier Magistretti, Soo Downe, Bengt Lindstrøm, Marie Berg, Katharina Tritten Schwarz

**Affiliations:** 1 Department for Social Management, Social Policies and Prevention, Center for Health Promotion and Prevention, Lucerne University of Applied Sciences and Arts, Lucerne, Switzerland; 2 Research in Childbirth and Health (ReaCH) Group, University of Central Lancashire, Preston, UK; 3 The NTNU Center for Health Promotion Research, NTNU, Trondheim, Norway; 4 The Institute of Health and Care Sciences, Sahlgrenska Academy, University of Gothenburg, Gothenburg, Sweden; 5 Midwifery, Health Division, Bern University of Applied Sciences, Bern, Switzerland

**Keywords:** Maternity care, salutogenesis, sense of coherence, tacit knowledge, health promotion

## Abstract

There is a lack of systematic evidence concerning health orientation in maternity practice in the current climate of risk avoidance. The midwifery professional project is orientated toward the preservation of normal physiological processes during the maternity episode. This study investigates accounts of midwives who were working in health-orientated birth settings, to examine if and how they frame a health orientation in professional practice. Twenty-seven narrative interviews were conducted with midwives working in pre-, peri-, and postnatal care in different maternity care settings in Switzerland, Austria, and Germany. In-depth and comparative pattern data analyses were conducted. The distinct practice orientation of the participants was revealed in three main concepts, underpinned by a common framework mirroring the three parameters of the Sense of Coherence (comprehensibility, manageability, and meaningfulness) described in Aaron Antonovsky's salutogenic theory. The midwives’ implicit salutogenic knowledge shaped their reported actions in supporting mothers, fathers, and families to have health-promoting experiences in maternity care. These results suggest that an implicit health orientation in maternity care practice can be prefered through examination of the practice reports of midwives working in settings that have a health-promoting philosophy. Implications for midwifery practice and research are discussed. Consideration is given to the relevance of the results for debates about avoiding overtreatment and for the operationalization of salutogenic theory in health care practice.

Health care systems in most countries at all resource levels are based on post-modernist cultural and societal paradigms, which have been conceptualized as risk avoidance (Beck, 1986) and acceleration (Rosa, 2013). The concept of acceleration describes the societal changes due to technical, digital, and economical progresses that result in accelerated rhythms of working and living (Rosa, 2013). The paradigms of risk avoidance and acceleration frame and construct societal norms, and those for health care and its sub-category, maternity care. This has resulted in harms as well as benefits for the health of mothers, babies, and their families (Downe, 2010). More recently, a turn toward so-called “salutogenic” health-promoting paradigms has been proposed, and the utility of this concept in explaining positive health outcomes has been subject to investigation to date in a range of health care contexts (Eriksson & Lindstrom, 2006, 2007). However, there is very little work in this area in terms of maternity care (Downe & McCourt, 2008).

The adverse effects of super-valuation of risk avoidance in maternity care have been discussed in terms of overtreatment, including the iatrogenic effects of overuse of episiotomy, caesarian section, induced and accelerated labor, and what has been termed more generally the “medicalization” of birth (Eriksson & Lindstrom, 2006). Acceleration processes and their consequences for maternity care have found specific scientific attention in the area of induced labor, demonstrating that the birth process itself and consequent postnatal care are being timed and shortened, in the name of risk avoidance and cost cutting (Moore & Low, 2012). A broad debate on over- and under-treatment in health care has been launched by the *British Medical Journal* (Moynihan, Heneghan, & Godlee, 2013) and by the New America Foundation (Saini, 2013). Participants in these debates are interested in rationalizing the use of interventions in health care in general, to improve health care and treatment, and to prevent a further rise in medical costs. A parallel development in maternity care has been a turn toward re-conceptualizing pregnancy and childbirth as largely a health-producing process rather than a risk-increasing one (Renfrew et al., 2013).

The midwifery profession, in particular, claims that it values a health orientation, and that midwives are the experts in promoting normal physiological pregnancy and birth (International Confederation of Midwives, 2011). However, despite a few studies that have examined how midwives do midwifery with healthy women and babies in the current health care climate in specific settings (Berg, Ólafsdóttir, & Lundgren, 2012; Byrom & Downe, 2010), little is known on this topic from a comprehensive contemporaneous cross-cultural perspective covering maternity care systems across more than one health setting and country. To the best of our knowledge, systematic studies linked to health theories and focused on comprehensive concepts of health-orientated practice in maternity care are lacking. Consequently, questions remain about how to define and put into practice health-orientated maternity care, and how to apply this knowledge to maternity care on a day-to-day basis. Judged by the mothers’ perspective, there are remarkable differences in subjective satisfaction between risk-oriented and health-oriented environments, especially with respect to the received psychological and emotional support (Meier Magistretti, Luyben, Villiger, & Varga, 2014; Thompson, 2011). Since findings from randomized trials showed benefits of health-oriented maternity care, after having ruled out selection bias, these differences in the *quality* of care remain unexplained. Questions remain about what midwives in midwife-led birth settings exactly do to provide the more satisfying emotional and psychosocial care reported by service users, and the better clinical outcomes that are seen in these trials. Identifying these phenomena could provide insights for the provision of improved care and outcomes for midwives and doctors working in settings that are associated with less positive findings.

An investigation of tacit knowledge could reveal some of the answers to these questions, for two reasons. First, any professional practice is linked to and rooted in tacit knowledge (Thornton, 2006). Second, psychosocial aspects of care, effective formation of interpersonal relationships, and health-orientated (salutogenic) approaches do not tend to be measured and counted in formal records or audits of terms. Moreover, professional expertise and tacit knowledge in maternity care have been widely debated and researched, but findings are limited to single characteristics of practice or to specific health issues (Berg et al., 2012; Downe, Simpson, & Trafford, 2007; Mann, 2005; O'Connell & Downe, 2009; Olafsdottir, 2006; Thompson, Hall Moran, Axelin, Dykes, & Flacking, 2013).

Consequently, questions remain unanswered about how to frame and how to put into practice a comprehensive health-oriented maternity care practice in various intercultural settings and how to link this tacit knowledge to existing health theories. This study, therefore, aimed to investigate whether midwives in three countries who were working in settings that identified themselves as having a health orientation did, in fact, hold a tacit comprehensive health orientation, and, if so, how they conceptualized and structured such an orientation in practice. In each case, the health-orientation status of the settings was confirmed by at least three authors of this article. The main questions of interest were:

- (How) do midwives express health-orientated concepts?
- (How) do they frame this health orientation in their practice?
- (How) can the description of a health-orientated practice be related to health theories?

## Methods

### Reflexive account

In line with emerging norms for the assessment of the rigor of qualitative research (Flick, 2009), all authors considered their reflexive standpoint in relation to this study. All authors have worked with and written about Antonovsky's theory of salutogenesis, and so all came to this study with pre-understandings about a certain way of perceiving health. CM is a clinical and health psychologist as well as director of research at her University. She has been engaged in practical, governmental, and research work in the areas of public health, health promotion, and prevention for more than 20 years. Therefore, she has an orientation toward interdisciplinary health approaches, including both medically or technically focused paradigms. SD is a trained midwife with 15 years of practice on an acute and busy labor ward. She has also spent 15 years in research, with a focus on normal(ising) pregnancy and childbirth. She is strongly committed to a health orientation in maternity care. BL a pediatrician has been clinically active for over 35 years, has specialized in Child Public Health but focuses on quality of life of children and families. BL in consultation with Aaron Antonovsky developed a deep understanding of salutogenesis and for 30 years has been involved in the international development of the salutogenic approach to health. MB has been a registered nurse midwife for 35 years and is a research professor in Health Care Sciences. Her area of work is reproductive and perinatal health and includes clinical practice as a midwife at a hospital-based antenatal care unit for pregnant women with complications. As a midwife, MB strives to maintain a health-orientated approach even when women are experiencing pregnancy complications. KT is a midwife with 25 years of experience in training midwives. She holds a master's degree in public health and is specialized in midwifery practical training and prevention of tobacco addiction among women. This research has been her first systematic work on salutogenesis.

### Participants

A purposive sample of 27 experienced midwives (professional practice ≥5 years) from organizations with a self-declared health orientation (hospitals for normal birth, birth centers, and independent midwives practicing home birth) from three different countries (Switzerland *n*=14, Austria *n*=8, and Germany *n=*2) were individually interviewed. Organizations were chosen by their self-declaration (organizations explicitly disclosing a health-oriented practice in their mission statements). Chosen organizations had to be approved by three local experts nominated by the authors’ group. Within the included organizations, midwives were sent an information letter from the authors. Midwives’ participation was voluntary, and they were explicitly advised that they could stop the interview at any point and that they could refuse to answer to any question they felt uncomfortable with.

### Measurement technique

Based on the assumption that methods of narrative interviewing and approaches of knowledge management such as storytelling are appropriate methodologies for elicitation of tacit knowledge (Linde, 2001; Thier, 2010), a narrative approach was chosen for this study. Specifically, an instrument was developed that included different prompts to stimulate reflection and disclosure from the participants. For instrument testing, individual pilot interviews with three retired midwives in the United Kingdom were conducted. These midwives had experienced (and themselves partially guided) a shift in maternity care from a strong medical orientation toward a health-orientated approach. The results of the pretest interviews were discussed by the authors, and with expert midwives and researchers at UCLAN^1^, after which a final interview schedule instrument was agreed upon.

### Interview procedure and data analysis

The interviews started with a broad opening question that asked each participant to recount one or more experiences that had marked her identity as a midwife. Depending on the answers, this question was followed up by related prompts based on the interview schedule. All the interviews were tape recorded and transcribed. After transcription, three interviews had to be excluded because they were conducted as discussions between the interviewer (a midwife) and the participant, rather than as an in-depth interview using the data collection instrument. Data analysis of the remaining 24 transcripts was conducted in two separate phases.

**Figure 1:**
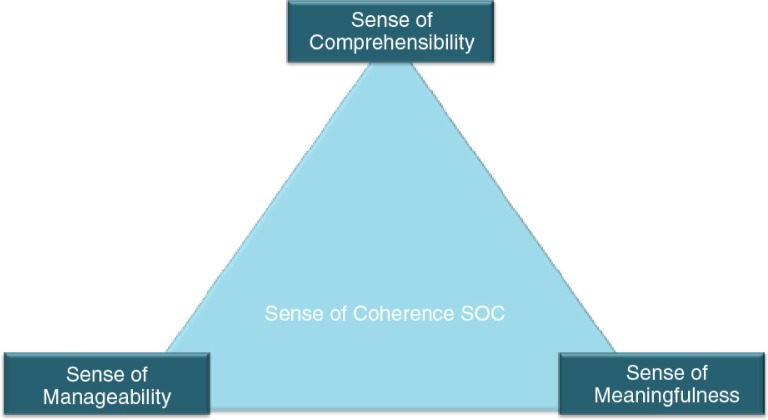
Dimensions of the Sense of Coherence (Antonovsky, 1987).

1. Phase one aimed to answer the question “do the participating midwives really have a tacit health orientation?” This consisted of a classical content analysis, in which all the passages of transcribed interviews that contained explicit statements related to health and health orientation were identified and categorized.
2. Phase two investigated how any tacit knowledge about health orientation was structured in the narratives. This analysis followed four steps. First, the transcripts were subject to a text analysis that discriminated narrative, descriptive, and argumentative sequences. Second, an inductive content analysis followed extracting the emerging topics of the text. Third, the topics were clustered into a graphic design showing the relevance, interaction, and structure of every topic in each interview. Fourth, the clusters were grouped by their inherent meaning following the method of pattern analysis (Kluge, 2000) (Figure 1). This phase included an investigation of the third study question, on how health-oriented practice can be related to health theories. Several theoretical approaches were tested to match the emerging analytic patterns in relation to pre-existing health theories. Five theories were tested: resilience (Zautra, Hall, & Murray, 2010), risk and protective factors (Lowdermilk, Perry, Cashion, & Alden, 2012), best practice framework (Broeskamp-Stone & Ackermann, 2010), health-promoting strategies (Nutbeam, 1998), and salutogenic Sense of Coherence (SOC) (Antonovsky, 1987). Cue categories were defined for each of the selected theories (see Table I) and used as the underpinning pattern for analysis. To ensure rigor, and given our strong pre-understandings related to salutogenic theories, we included searches for disconfirming data into our analysis at this stage (Table I).

**Table I:** Theories and related categories used for testing pattern analysis.

Theoretical framework	Selected categories for pattern analysis
Resilience	Selected factors of resilience in birth contexts: - Active coping - Realistic and flexible attribution - Positive social support - Optimism - Positive self-concept
Risk and protective factors	- Avoiding risk factors (e.g., smoking in pregnancy, domestic violence, poor diet) - Strengthening protective factors (e.g., social support, expected competence as a mother)
Best practice framework	Knowledge - Scientific knowledge of determinants and effectiveness - Knowledge from experts gained in practice Values - Health equality - Sustainability - Transparency Context - Social, legal, political context on regional, local, and institutional levels
Health-promoting strategies	Adopted strategies of - Empowerment - Healthy environments/settings - Participation
Salutogenesis	Sense of Coherence: - Sense of Comprehensibility - Sense of Manageability - Sense of Meaningfulness

It was agreed that an effective theoretical analysis would mean that the candidate theory should explain all or most of the narratives.

## Results

The country of residence and area of practice of the participants are presented in Table II.

**Table II:** Country of residence and area of practice of participants.

Total number of participants, *n*=24			

Country		Working in	
Switzerland (S)	*n*=14	Antenatal care	*n*=4
Austria (A)	*n*=8	Perinatal care	*n*=16
Germany (G)	*n*=2	Postnatal care	*n*=4

## Summary findings

The tests on five selected transcripts showed four of the theories accounted for less than half of the interview narratives, namely resilience (Zautra et al., 2010), risk and protective factors (Lowdermilk et al., 2012), the best practice framework (Broeskamp-Stone & Ackermann, 2010), and health-promoting strategies (Nutbeam, 1998). In contrast, salutogenic theory was able to explain most of the narratives through the dimensions of the Sense of Coherence described by Antonovsky (1987). Even though density, interaction, and differentiation of the narrative clusters varied in the interviews, the SOC meta-pattern covered almost every detected cluster, and provided a distinct description of how midwives actually foster the senses of comprehensibility, manageability, and meaningfulness. As noted above, given the strongly consistent pre-understandings of the authors, the technique of iterative analysis was employed, looking for theoretical saturation and concurrent attention to disconfirming data, to ensure that the emerging findings were not constrained by the beliefs and values of the authors.

The SOC is “a global orientation that expresses the extent to which one has a pervasive, enduring though dynamic feeling of confidence” (Antonovsky, 1987, Figure I) that

- Things happen in an orderly and predictable fashion and a person can understand events in life and reasonably predict what will happen in the future. (Sense of Comprehensibility)
- One has the skills or ability, the support, the help, or the resources necessary to take care of things, and that things are manageable. (Sense of Manageability)
- Things in life are interesting and a source of satisfaction, things are genuinely worthwhile, and there is a good reason or purpose to care about what happens. (Sense of Meaningfulness)

**Figure 2:**
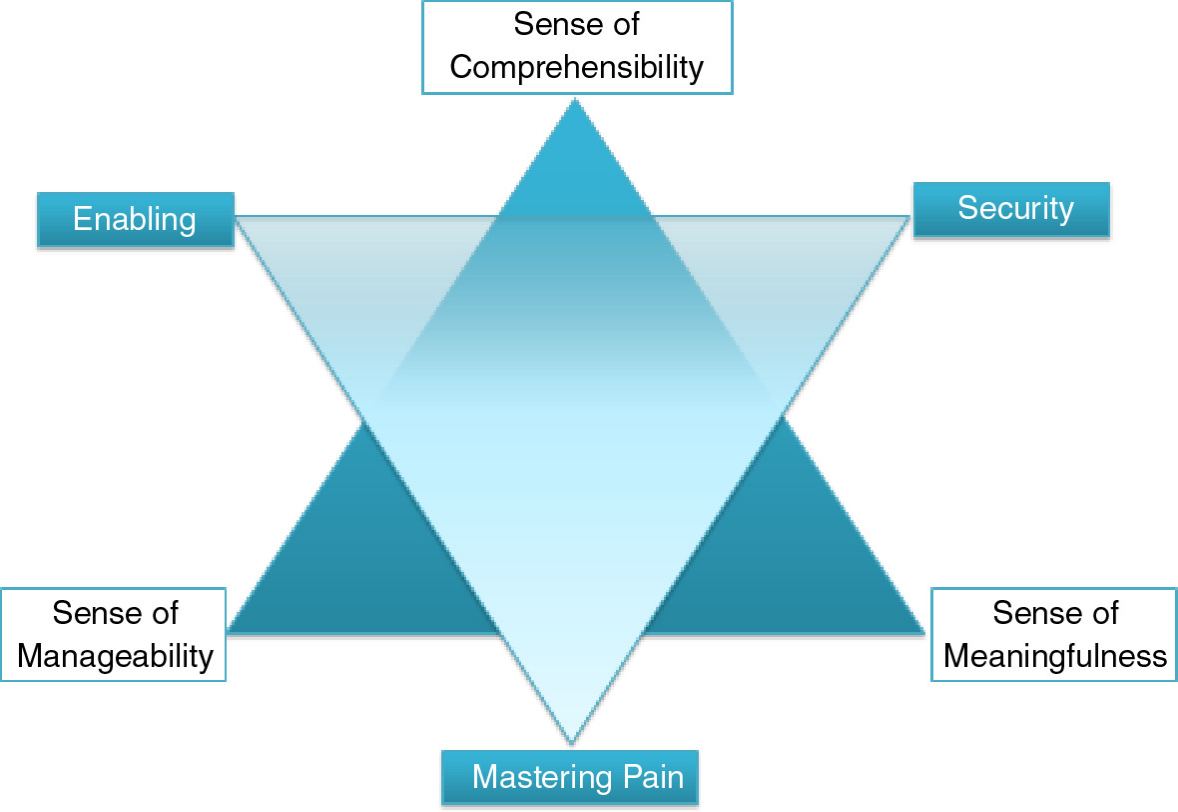
Dimensions of the Sense for Coherence in health-oriented midwives’ narratives.

Each of these dimensions was elaborated in the participants’ narratives as will be shown in the detailed findings sections, which are presented according to the research questions and ordered as A, B, and C. Figure 2 provides an overview of the final six dimensions of midwives’ conceptualizations described below:

## Question A: How did the participating midwives express health-orientated concepts?

Three narrative themes emerged from the analysis of the narratives under the heading of “implicit health orientation.” The first concerns health explicitly. These comments characterized practice as part of health promotion, pointing at its relevance for the lifelong health of mothers, babies, and families:

To me, pregnancy and birth don't only matter now … I want to maintain the health of the woman and the baby … I want to think of their next 20, 30 years … it's now that we're setting the stage for health. (Int.3)

Thereby, respondents emphasized the woman's and the family's well-being:

It isn't the point to put your ideals of a good birth on the woman. The real issue is that the woman has a good experience regardless if she has had a Caesarian or a PDA^2^ or a completely natural birth … the aim has always to be to have a happy family. (Int. 7)

It is good when the woman feels good after birth, no matter if she has had a Cesarean or a spontaneous birth. (Int. 8)

The narratives of the second theme reflect a health orientation in contradistinction to pathological concepts. In this pattern, respondents related what they did not want to do (any more) and they explained their orientation in contrast to a pathologically or technically oriented practice.

It was a machine: Women came in, they were treated and they left … birth lost its meaning—it was like eating popcorn. (Int. 7)

Care is difficult, because we are too few professionals and we have to look after too many women….we can really met their needs cause we are too few, we are overstrained and we lack time. (Int. 8)

In the third emerged theme health is conceptualized implicitly, rather than in explicit health terms, by speaking of overarching concepts such as “spiritual birth,” “natural birth,” or “loving relationships.”

At times, birth is considered an illness. It is our task to show it is a natural process. (Int. 12)

It is when the women's individuality gets lost, when every woman has the same system to have to adapt to. (Int. 10)

Some respondents didn't express direct statements but used examples to illustrate what was not, in their view, a health-orientated birth practice:

Of course we have a birthing pool, but it is in the most uncomfortable room, a room with a bad atmosphere, you wouldn't like to enter, and the doctors don't really support—so it's difficult to work. (Int. 4)

In the meantime I know I can't trust these common-or-garden diagnoses—I have to base [decisions] on my experience. (Int. 5)

Most respondents described health orientation in maternity care as a function of their mutual interaction with the woman giving birth.

We could create a relaxed atmosphere, and the woman could breathe and I helped her to breathe and I was just there. But I felt connected to the women's spirit and it was a very special atmosphere and it was of this energy that this birth emerged. (Int. 4)

## Questions B and C: How did participating midwives frame health orientation in their practice, and how can this be related to health theories?

### The Sense of Comprehensibility in midwifery practice

Midwives’ narratives in this study showed a concern for fostering comprehensibility in maternity care. Comprehensibility is described as a process of actively investing in understanding the woman, providing orientation and security, and enabling the woman to self-manage her pregnancy, her birth process, and the postnatal period.

#### Active investment in understanding the woman

Midwives described the process of understanding the women as an *active investment* in comprehension through interaction, communication, perception, and observation. They did not supervalue diagnostic instruments and checklists: they used them because they were required to, but real understanding of the condition and situation of the mother and fetus/baby took place through other strategies. Respondents reported ongoing processes of continuous interaction and communication with the women. In a repeated process of feedback loops, participants verified their observations by reflecting back to the woman what she had said in order to precisely capture the woman's situation, needs, and objectives.

Respondents also gave evidence of how they refined their skills of perception, observation, and embodied communication within this process in a comprehensive way that covered general observation as well as various ways of perception.

I listen to the breathing, I smell the stool, I observe the sweat and her position. (Int. 4)

Participants reported that they actively used irregular and potentially irritating requests and demands from the woman they encountered as prompts to try to understand her point of view, and that they consciously intended not to impose their own values on her:

You know, then I got the entry form, and I read that she wanted an early PDA^3^. Then I didn't think, “Oh God, there's one who wants an early peridural anaesthesia!” Then I said, There's always a reason why she wants this. And then I went to ask her, just because I was interested, I wanted to understand. (Int. 7)

#### Providing orientation

Participants consciously provided orientation to give the woman an opportunity to “manage the chaos.” They helped the woman to understand what she was experiencing and what awaited her. They put a distinct emphasis on simple, repeated, and re-repeated explanation in order to make sure that the woman and the couple truly understood what was happening. They described *how* they explained and they reported *what* they explained:

You also have to explain technically, explain the advantages and disadvantages of something you recommend … the woman has to know … when I am aware of what I am doing, the woman knows she can rely on me, she can trust me. (Int. 7)

Beyond the information about what was occurring at a given moment, participants provided orientation for what was to come in order to render the situation foreseeable. Concurrently, they reported providing implicit orientation in the sense that they structured the situation and the interaction:

When I know I have a meeting with a woman who needs more time than the usual 45 minutes we have, then I invite her at off-peak hours to be free to listen to her or to give her the chance to bring somebody along who is important to her. This has to have space, this always has to have space. (Int. 21)

#### Providing security

Security was conceptualized as a result of understanding the woman and providing orientation. Some midwives framed security as a matter of space given and as an atmosphere they created:

This was my task with her: I wanted to give her my presence, to provide a framework for her … to give her the security that allows her to go with the birth process. She has to know, “I have a safe and intimate framework…and I have somebody who observes, who cares and who looks after me.” (Int. 26)

Security was maintained even if difficulties occurred:

I try to talk in positive terms. I don't want her to have fear or to feel unsafe. She has to feel that I am assuming my responsibilities and that I'm always honest about the situation she and the baby are in. (Int. 15)

#### Enabling the woman

The concept of “enabling the woman” was reported in all the narratives. It can be described as the activities that strengthen the women's physical and emotional conditions to enable them to go with the process of pregnancy, birth, and the postnatal period. The respondents fostered a positive bodily perception. They reported that they advised each woman to learn from her own body, from her embodied sensations, and to sense and to touch themselves:

I told her, “Birth doesn't happen in your head. It is a bodily process.” And I tried to tell her, “You needn't read much, it's better for you to do breathing exercises or practice yoga and talk to your baby. You discuss your fears with the baby or you tell him: We will make it, the two of us together.” So I gave her some ideas and at the end she really managed so much better. (Int. 3)

The women were also encouraged in their ability to discover their own knowledge and find their own solutions. Women were given time to reflect and to become familiar with the decisions taken. Participants stressed the importance of asking the woman if she felt comfortable with a chosen decision.

I just put the same question several times in different ways. If her answers are similar every time, then I can be sure that it is all right for her … many women are not used to having the opportunity to talk—they are used to giving short answers to questions like—“Do you have any problems with your legs?” “Yes or no.” (Int. 18)

I was the only one to tell her she was right when she didn't want to put her baby in day care since he cried every time she left him. She then asked her parents to come for one day a week – and now everything is fine and she is so happy. (Int. 17)

Trusting a woman's own perception sometimes meant allowing her to make choices that are commonly not considered ideal:

She just had a wonderful birth process. But she pushed lying on her side. I decided to wait—and really, she gave birth lying on her side and everything went so fine and it was so beautiful. (Int. 19)

### The Sense of Meaningfulness in midwifery practice

The Sense of Meaningfulness consists of the factors enabling the women to meet the challenges of pregnancy, birth, and childbearing. Midwives’ narratives revealed two major concepts of the Sense of Meaningfulness. The first implies that motivation is inherent to the birth process itself, while the second affirms that the commitment to a specific target expresses motivation in practice.

#### Inherent meaning of pregnancy and birth

Midwives described birth as an elemental force activating an innate primordial trust in the woman. The expressions midwives used to describe this power varied, including “holy moments,” “grace,” “beauty of pregnancy and birth,” “joy,” “optimism,” and “humor.”

Most midwives’ statements concerning the Sense of Meaningfulness were mentioned in the reflective comments at the end of the narrative sequences, echoing the stories they told:

It's our task to mobilize the power of the woman and to bring her in contact with her elementary power to give birth, to teach her to trust it and to demonstrate to her that her power is useful, that it does make sense and even that it has a deeper sense. (Int. 19)

Participants talked about strengthening the innate power and trust of the woman, mainly by giving space to it, by offering a framework without external disturbance, by accepting and welcoming the woman in her uniqueness, by giving confidence, and by creating an atmosphere in which the woman or the couple felt at ease. They reported being something like a mindfully focused presence that included at times holding back personal attitudes or preferences so as to facilitate the woman's contact with her own power.

#### Target-oriented meaning of pregnancy and birth

The target-oriented meaning revealed by the midwives’ narratives emphasized the relevance of will and determination as well as the active orientation toward a chosen or desired aim in pregnancy and birth. It includes two sub-concepts. One states the importance of a positive birth experience for the health and well-being of the woman, the baby, and the family, while the other underlines the importance of determination, will, and investment if a certain aim has been set (e.g., a normal or a water birth).

This musician, he knew the child was breech—and he just didn't stop teasing and tickling—he didn't stop—up to the point when the baby turned around. I was surprised, really surprised. (Int. 5)

#### Lack of meaning in pregnancy and birth

A few narratives show poorly elaborated clusters concerning the Sense of Meaningfulness or even lack these clusters completely. These narratives reveal another common pattern instead: they contain detailed and elaborated clusters describing adverse working conditions or reporting personal resignation and the wish to quit the job. This was contextualized usually by a lack of Sense of Manageability.

### The Sense of Manageability in midwifery practice

The concept of manageability was more likely to be expressed as a function of the presence or absence of conflict experienced by the participants in their everyday practices. As such, it seemed to be the dimension that was most vulnerable to context. On reflection, this makes sense since manageability is about how one deals with the actual process demanding great flexibility and readiness for change factors. Contrary to the dimensions of comprehensibility and meaningfulness, the Sense of Manageability was reported indirectly in terms of the contrasting paradigms of technical risk avoidance and health orientation. Their narratives revealed a constant dilemma that midwives experience on three levels of conflict: organizational, inter-professional, and intra-personal.

#### Organizational conflicts

On an organizational level, participants contrasted the liberty of action, the cooperation in teams, and the subjective well-being in health-oriented environments to the opposite conditions in traditional hospitals, mentioning strict hierarchies, extensive regulations, and excessive demands by the number of women to be responsible for.

In hospital, everything went in a medical order—you just had to consider the medical aspects and now, I really have the sense/feeling of being a real midwife and being able to care for the woman. (Int. 24)

Consequently, detailed descriptions were found of the quality of care the women received. Participants contrasted individual and empathetic care in a process of shared responsibility in health-oriented settings to directed standard procedures in impersonal and fragmented treatment in hospitals.

After the third birth that day, I just lacked sensitivity. I didn't listen as carefully as I would have liked. And then I told her what she had to do instead of giving her three options to choose … I was directed by my opinion and not open to her wishes. (Int. 26)

#### Inter-professional conflicts

A second group of narratives described the conflict of paradigms as a conflict among professionals. They described several situations of rivalry and competition between “doctors” and “midwives.”

Ah, I was proud and this was my triumph—when the head physician did the control and had to admit that I was right—he thought that the situation was past hope, but I made it. (Int. 9)

#### Intrapersonal conflicts

A third group of narratives represented the conflict of paradigms as an inner conflict of midwives’ personal values and as persistent cognitive or emotional dissonances. These midwives framed the conflict of paradigms as their personal problem and as an inevitable part of their professional role:

We had to concentrate on the facts, the ones used in Germany, you know. But there was irrationality in it. The doctor indeed, she insisted that the child wasn't big. What she said was complete nonsense…looking back we should have done the Caesarian much earlier. But they kept insisting that the child wasn't big. (Int. 6)

I went home after hospital service and I was stressed and frustrated. And I thought, “What have I done?” I left the hospital and I was relieved that luckily nothing bad had happened. And I always had this feeling of “What did I do?” I just could do what was medically necessary there, just the medically necessary. (Int. 14)

#### Overcoming the conflicts of manageability: bridges to the Sense of Meaningfulness

Some participants found ways to overcome the conflicting paradigms by focusing on broader concepts, such as the well-being of the woman. They stated that as long as the woman's well-being is aimed at, “anything goes” be it a technical intervention or a normal birth. Other respondents reported a broadening “if…then” strategy, assuming that a health-oriented process is only possible if midwives succeed in establishing a good relationship, an adequate bonding, a spiritual connectedness, or a relationship of humanity and love with the women. To reach these conditions, midwives and couples have to develop a strong will and a clear commitment. Thereby the women succeed more easily in managing the process and difficulties such as pain lose significance. Consequently, midwives reported that the experience of good self-management improves motivation, which facilitates the process and thereby reinforces motivation in a self-enhancing positive process:

I told her, “See, it hurts now and you would prefer to go home or tell me to just finally do something! But your pain makes sense, you will have a real benefit from it, you will see that the baby will make you happy. It does make sense”…and by this, we could turn the tide and the process went well. (Int. 7)

The concept of “enabling the woman” seems to be the bridging element linking the Sense of Comprehensibility with the dimension of manageability:

## Synthesis: *Health-orientated midwifery practice and SOC*

The SOC for midwifery practice can be conceptualized as follows, based on the analysis of the midwives’ narratives above. It includes:

- The Sense of Comprehensibility as a dynamic and interactive cycle of comprehensive understandings and explanations
- The Sense of Meaningfulness as the trust in either the innate power of birth or as the will to realize a targeted aim
- The Sense of Manageability as the ability to be with the woman while managing contrasts and conflicts of current paradigms in professional contexts

In detail, the participants’ reports suggest that midwives who are operationalizing the midwifery project in practice foster comprehensibility by facilitating the understanding of the needs and resources of women, explaining processes and procedures. Thereby they provide orientation and security, enable women to make their own choices, and constantly return to these processes in the dynamic context of each woman's pregnancy, birth, and postnatal period. The midwifery Sense of Meaningfulness seems to include both intrinsic and aim-orientated forms of motivation. In contrast, midwifery manageability remains in a state of tension, held between contradictory concepts of contemporary maternity care. The capacity to realize midwifery meaningfulness and comprehensibility depends on the capacity of the midwife to resolve these tensions. The three dimensions of the SOC (comprehensibility, manageability, and meaningfulness) are strongly interconnected. Comprehensibility and manageability are linked by the concept of enabling the woman, while comprehensibility and meaningfulness are connected by the concept of security. Moreover, there is a concept of “mastering” describing a self-enhancing process between manageability and meaningfulness in that the experience of successful manageability fosters motivation and vice versa.

## Discussion: The relevance of midwives’ Sense of Coherence

In the present study, we have demonstrated that tacit knowledge of health-orientated maternity care can be revealed by the chosen narrative approach. Following the methodology of storytelling analysis (Thier, 2010; Thier & Erlach, 2005), we can state that the narrative parts of midwives’ statements depict their action and reveal their tacit knowledge, whereas the observing and interpreting statements mirror attitudes and subjective concepts. The description of the Sense of Comprehensibility and the ones of the Sense of Manageability as well as the bridging concepts are based on narrative texts. The concept of the Sense of Meaningfulness additionally includes observations and interpretations, since this dimension comprehends elements of subjective sense-giving and reflection.

Limitations of the study include the specific sampling strategy. Although the sample included midwives from three countries, from a range of practice settings, and from those working in pre-, peri- or postnatal care, the participants were selected purposively, and they were all based in European countries with a strong tradition of midwifery practice, so the findings do not necessarily apply to all practicing midwives in all settings.

With these caveats, we found that the midwives participating in the study expressed a general health orientation either in deictic or in delimiting statements. In the deictic concepts, they stated to consciously address women's and family's health in their daily practice or they framed health by related subjective concepts such as “natural” or “loving” practice in maternity care. Midwifes’ delimiting statements consisted in what they said they didn't do (and didn't want to do) in their daily practice, mainly opposing a technological or a pathological orientation of maternity care. These findings correspond to results of prior researches (Davis, 2010).

Structure analysis of the narratives in this study showed an accurate correspondence and a practical extension to Aaron Antonovsky's concept of the SOC and to its main dimensions of comprehensibility, manageability, and meaningfulness (Antonovsky, 1987). Midwives participating in this study fostered comprehensibility by facilitating the understanding of the needs and resources of women, explaining processes and procedures, providing orientation, and enabling women to make their own choices. In this study, midwives’ concept of security associates the Sense of Comprehensibility to the Sense of Meaningfulness confirming similar findings on the importance of security and trust (Nilsson, Thorsell, Hertfelt Wahn, & Ekström, 2013). Corresponding to previous studies (Walsh & Devane, 2012), this study illustrates, salutogenic manageability is feasible if midwives succeed in “following the women” despite conflicting philosophies and demands in their work environment (Blaaka & Schauer, 2008). Both the contradictory concepts of risk avoidance and health orientation as well as the concept of “being with the woman” have been described by other authors (Berg et al., 2012; Olafsdottir, 2006). The motivational dimension of the SOC, captured in the Sense of Meaningfulness, could be related to previous studies on midwives’ values and orientation (Byrom & Downe, 2010).

Midwives’ SOC gives a scientific voice to health-orientated tacit knowledge that so far has been silenced in the definition of professional quality and standards in maternity care. Even though health-orientated practice has been previously described for specific situations, tacit knowledge has hardly ever been linked to health and health theory. Therefore, this study contributes to close the gap between the knowledge generated in studies of evidence-based maternity care for defined procedures in midwifery practice and the many undescribed (or partly described) situations, where practice of maternity care is ill-defined, complex, and highly individual (Finlayson & Downe, 2013). Linking maternity care practice to salutogenic theory by clearly defined concepts could allow a consistent theoretical description integrating priorly researched single concepts such as relationships (Macpherson, Roque-Sanchez, Legget, & Segarra, 2016), joyfulness (Cottrell, 2016), or trust (Nilsson et al., 2013). Conclusively, the kaleidoscopic character of midwifery practice (Borelli, Spiby, & Walsh, 2016) could be linked to empirically accessible concepts like the Sense of Coherence.

This study has shown clear linkages to the concept of the SOC in salutogenic theory: the underlying concepts of the SOC revealed in this study open far-reaching practical impacts, since a strong SOC has been shown to be crucial for the immediate and lifelong health of mothers, babies, and families, as well as for the parent's abilities to deal with the illness, crisis, and handicaps of their children (Lindström & Eriksson, 2010). Moreover, tacit knowledge of fostering health in maternity and early-life care might bring insights into to the debates about overtreatment and medicalization, showing that there is an alternative to risk avoidance and economic acceleration that does not replace the ruling paradigm, but that can work alongside it. The debate about “avoiding avoidable care” has recently been developed into a discussion of the importance of the “right care” (Saini, 2013) and of the need to promote health more rigorously (Bortz, 2011). The findings of this study may form a basis for future studies that seek to promote such a turn in maternity care provision specifically, and in health care provision in general. Identification and removal of the manageability tensions could help in this shift. More immediately, this study's results may contribute to an explanation of the outcome benefits (Hodnett, Downe, & Walsh, 2012) and the higher psychosocial well-being of mothers (Meier Magistretti et al., 2014) provided by maternity care that is based on a philosophy of maximizing well-being, rather than minimizing risk.

This study was limited to midwives, since there had been strong arguments to assume a predominant health orientation in this professional group. Nevertheless, some obstetricians, nurses, and other health professionals in maternity care as well as in other medical fields also practice on the basis of a positive health orientation. To encourage “next medicine” (Lindström, 2012) and to refine the discourse on “avoiding avoidable care,” it will be fruitful to do similar studies involving various professions in various fields of health and of medical care.

## Conclusion

This article provides a theory-based description of health orientation in the practice of midwives. It renders health-orientated and health-promoting practice visible and accessible for initiatives aiming at improving maternity care. The findings also open broader opportunities for teaching and education. The extent to which the practices described in this research actually improve health in women and babies has still to be demonstrated in future research examining the mechanism of effect for “what works.” Salutogenesis reflects a life process perspective, dealing with factors that explore and develop the capability of both individuals and systems. For maternity care, this includes the process of birth itself and all the actors involved—both lay men or women and professionals. Ideally, the health system is designed to support and develop the SOC of the women and families who are trying to achieve the best possible birth process. To be able to do this in an optimal way, a deep understanding of the needs of those giving birth (women and families), optimizing best professional knowledge and skills to manage child birth, and optimal application of best professional knowledge, skills, and values are required. A maternity service in which these attributes can flourish would operationalize a “sense FOR coherence” (Lindström & Eriksson, 2010) that has been described as a “a sense of how to improve the sense of coherence of the people we work for” (Koelen & Lindström, 2016, p. 34). This concept could usefully frame a move toward prioritization of health both for the contemporary maternity field, and, more broadly, for medical care in general. Thus this article can serve as a guide for other professionals. It also serves as a gate opener for the much needed further development of the salutogenic model in practice.

## Notes

1 An EU Cost Action short-term scientific mission (STSM) at UCLAN University in Preston, Lancashire, England, was conducted within COST-Action IS0907 to develop and pretest the instrument.

2 Peridural anaesthesia.

3 Peridural anaesthesia.
